# Clemastine and metformin extend the window of NMDA receptor surface expression in ageing oligodendrocyte precursor cells

**DOI:** 10.1038/s41598-024-53615-x

**Published:** 2024-02-19

**Authors:** Yasmine Kamen, Kimberley Anne Evans, Sergey Sitnikov, Sonia Olivia Spitzer, Omar de Faria, Mert Yucel, Ragnhildur Thóra Káradóttir

**Affiliations:** 1 Cambridge Stem Cell Institute and Department of Veterinary Medicine, University of Cambridge, Jeffrey Cheah Biomedical Centre, Puddicombe Way, Cambridge Biomedical Campus, Cambridge, CB2 A0W UK; 2 Department of Physiology, BioMedical Center, Faculty of Medicine, University of Iceland, Reykjavik, Iceland

**Keywords:** Oligodendrocyte, Neural ageing, Ion channels in the nervous system

## Abstract

In the central nervous system, oligodendrocyte precursor cells (OPCs) proliferate and differentiate into myelinating oligodendrocytes throughout life, allowing for ongoing myelination and myelin repair. With age, differentiation efficacy decreases and myelin repair fails; therefore, recent therapeutic efforts have focused on enhancing differentiation. Many cues are thought to regulate OPC differentiation, including neuronal activity, which OPCs can sense and respond to via their voltage-gated ion channels and glutamate receptors. However, OPCs’ density of voltage-gated ion channels and glutamate receptors differs with age and brain region, and correlates with their proliferation and differentiation potential, suggesting that OPCs exist in different functional cell states, and that age-associated states might underlie remyelination failure. Here, we use whole-cell patch-clamp to investigate whether clemastine and metformin, two pro-remyelination compounds, alter OPC membrane properties and promote a specific OPC state. We find that clemastine and metformin extend the window of NMDAR surface expression, promoting an NMDAR-rich OPC state. Our findings highlight a possible mechanism for the pro-remyelinating action of clemastine and metformin, and suggest that OPC states can be modulated as a strategy to promote myelin repair.

## Introduction

In the central nervous system (CNS), oligodendrocyte precursor cells (OPCs) give rise to oligodendrocytes which provide metabolic support to neurons and make myelin, a lipid-rich membrane that is essential for rapid electrical conduction and input synchronization^1,2^. OPCs proliferate and differentiate into myelinating oligodendrocytes throughout life^3,4^; however, OPCs’ proliferation and differentiation potential declines with age, coinciding with the onset of neurodegenerative disorders and cognitive decline^5–7^, and remyelination failure^8^. Hence, therapeutic strategies to enhance remyelination have focused on identifying compounds such as clemastine and metformin to promote OPC differentiation or rejuvenate OPCs to overcome the age-related block in differentiation^9,10^.

Many cues are thought to regulate OPC proliferation and differentiation, including neuronal activity, which OPCs can sense and respond to through their voltage-gated ion channels and glutamate receptors. However, OPCs are regionally and temporally diverse^11–13^, first appearing as a homogeneous population lacking voltage-gated ion channels or glutamate receptors, and gradually acquiring voltage-gated Na^+^ channels (Na_V_), voltage-gated K^+^ channels (K_V_), AMPA/kainate receptors (α-amino-3-hydroxy-5-methyl-4-isoxazole propionic acid; AMPARs/KARs), and NMDA receptors (*N*-methyl-d-aspartate; NMDARs) at different rates in different regions^13^. The temporal and regional surface expression patterns of these channels correlate with the reported diversity in proliferation and differentiation potential of OPCs^4,14,15^, suggesting that this electrophysiological diversity might indicate dynamic cell states^16^. For instance, the proportion of OPCs with NMDARs, which underlie activity-dependent myelination and remyelination^17^, peaks at the time of highest myelination in the forebrain, and NMDARs are no longer detected in the parenchymal forebrain by 9 months, at which age remyelination potential has declined^13^. Hence, one possibility is that pro-remyelination compounds such as clemastine and metformin may promote a particular OPC state that is primed for differentiation^16^.

Here, we use whole-cell patch-clamp to test whether clemastine and metformin promote a specific OPC functional state, as identified before^13,16^. We find that clemastine and metformin modulate ion channels and glutamate receptors in OPCs, extending the window of NMDAR surface expression. Our results highlight a potential mechanism for the pro-remyelinating action of clemastine and metformin, and suggest that altering OPCs’ sensitivity to neuronal activity by modulating cell states may be a mechanism to promote remyelination.

## Results

### Clemastine extends the window of NMDAR surface expression

**Figure 1:**
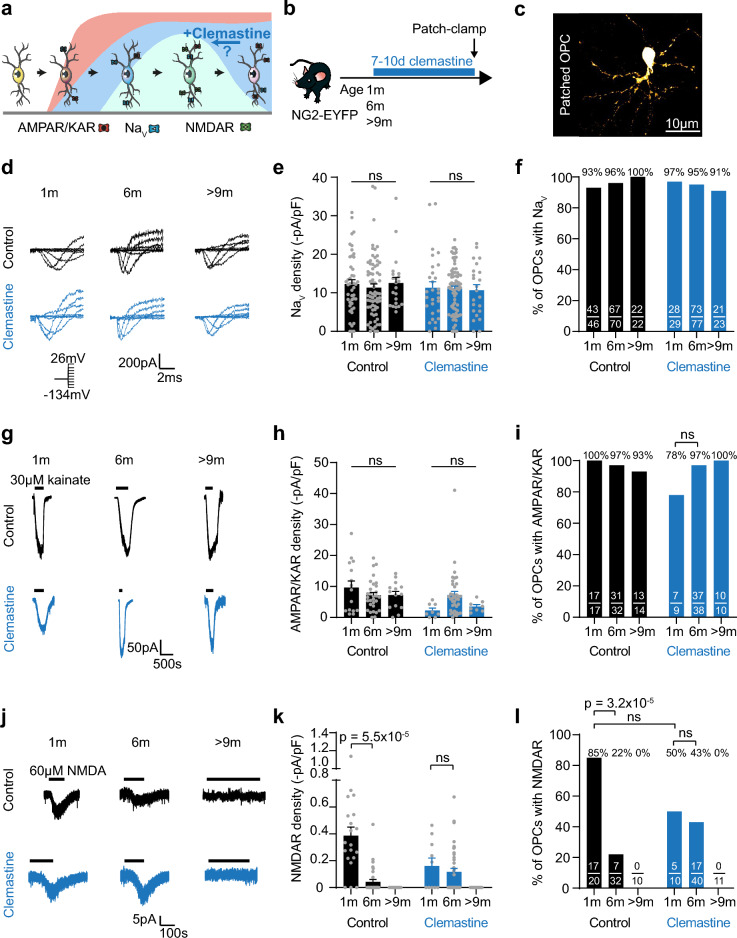
Clemastine extends the window of NMDAR surface expression. (**a**) OPCs acquire different voltage-gated ion channels and glutamate receptors with age, with at least five different electrophysiological profiles identified by Spitzer et al.^13^. The curves represent the proportion of OPCs with AMPARs/KARs (red), Na_V_ (blue), and NMDARs (green) with age. Adapted from Spitzer et al., Neuron 2019^13^ with permission, CC BY. (**b**) 1 m, 6 m or > 9 m old NG2-EYFP mice were administered clemastine in drinking water for 7–10 days before patch-clamp recordings. (**c**) OPCs were whole-cell patch-clamped and dye filled with Lucifer Yellow. (**d**) Leak-subtracted traces of Na_V_ currents in response to 20 mV steps from a holding potential of − 74 mV (inset, voltage steps from − 134 to + 26 mV) in cortical OPCs from control (black) and clemastine-treated (blue) 1 m, 6 m or > 9 m mice. (**e**) Na_V_ current density did not differ with age in adult mice, and was unaltered by clemastine administration. Control and clemastine-treated OPCs across ages, two-way ANOVA: condition main effect: p = 0.3; age main effect, p = 0.8; interaction, p = 0.8, indicating no difference in density between conditions, nor with age. 1 m control: n = 46, 6 m control: n = 70, > 9 m: n = 22, 1 m clemastine: n = 29, 6 m clemastine: n = 77, > 9 m clemastine n = 23. p values on the graph indicate the age main effect from the two-way ANOVA. (**f**) The proportion of OPCs with Na_V_ did not differ with age in adult mice, and was unaltered by clemastine administration. (**g**) 30 µM kainate-evoked currents in cortical OPCs from control (black) and clemastine-treated (blue) 1 m, 6 m or > 9 m mice. (**h**) AMPAR/KAR density did not differ with age, but was lower in clemastine-treated mice. Control and clemastine-treated OPCs across ages, two-way ANOVA: condition main effect: p = 0.008; age main effect, p = 0.4; interaction, p = 0.04, indicating a difference in density between conditions, but not with age. 1 m control: n = 15, 6 m control: n = 30, > 9 m: n = 14, 1 m clemastine: n = 8, 6 m clemastine: n = 37, > 9 m clemastine n = 8. p values on the bar graph indicate the age main effect from the two-way ANOVA. (**i**) The proportion of OPCs with AMPARs/KARs did not differ with age, nor with condition (all ages pooled, control: 96.83%; clemastine: 94.74%; p = 0.9, χ^2^). The proportion of OPCs with kainate-evoked responses did not differ between 1 m clemastine-treated and 6 m clemastine-treated mice (χ^2^; p value on bar graph). (**j**) 60 µM NMDA-evoked currents in cortical OPCs from control (black) and clemastine-treated (blue) 1 m, 6 m or > 9 m mice. (**k**) NMDAR density decreased with age in control animals. Clemastine administration prevented this decrease in NMDAR density in 6 m old mice, but did not prevent the loss of NMDARs in 9 m old mice. Control and clemastine-treated OPCs at 1 m and 6 m, two-way ANOVA: condition main effect, p = 0.07; age main effect, p = 7.9 × 10^–6^; interaction, p = 4.5 × 10^–4^, indicating no difference in overall density between conditions, but a difference in the age-driven decline of NMDARs in OPCs with clemastine treatment. 1 m control: n = 20, 6 m control: n = 32, 9 m control: n = 10, 1 m clemastine: n = 10, 6 m clemastine: n = 40, 9 m clemastine n = 11. p values on the bar graph were calculated with Holm–Bonferroni post-hoc tests. (**l**) The proportion of OPCs with NMDARs decreased with age in control animals. Clemastine prevented this decrease in 6 m OPCs, but did not reverse the loss of NMDAR at 9 m. p values are from χ^2^ tests. Data for (**e**,**h**,**k**) are shown as mean ± SEM, with grey dots indicating individual recorded cells. The numbers on the bars in (**f**,**i**,**l**) indicate the number of responding cells over the number of recorded cells.

Clemastine, a muscarinic receptor antagonist, promotes differentiation and remyelination in murine demyelination models and showed promising outcomes in a remyelination clinical trial^9,18^. However, it is not clear how clemastine enhances OPC differentiation. As neuromodulator signalling can modulate glutamate receptors and ion channels in neurons and cultured OPCs^19–21^, we asked whether clemastine promotes remyelination by altering OPC membrane properties and promoting a specific OPC state (Fig. 1a).

To investigate this, we used heterozygous NG2-EYFP knock-in mice (NG2-EYFP); in these mice all parenchymal EYFP positive cells are OLIG2 and NG2 positive throughout life, indicating that the EYFP expression tightly follows the expression of the NG2 protein^22^. We administered 20 mg/L clemastine in the drinking water of 1 month old (1 m), 6 m, or > 9 m NG2-EYFP mice for 7–10 days and whole-cell patch-clamped NG2-EYFP^+^ OPCs in the cingulate, motor, and somatosensory cortices (Fig. 1b,c). We detected no change in water consumption between control or treatment groups. In control animals, passive membrane properties including membrane resistance, the slope of the inward rectifying K^+^ current (inward conductance), and resting membrane potential did not significantly differ after 1 m in cortical OPCs (Supplementary Fig. S1). Clemastine administration did not alter these passive membrane properties in OPCs much, with membrane resistance (p = 0.3), inward rectifying K^+^ conductance (p = 0.1) or resting membrane potential (p = 0.2) similar between conditions (two-way ANOVA, condition main effect; 1 m control: n = 16, 6 m control: n = 49, > 9 m control: n = 9, 1 m clemastine: n = 22, 6 m clemastine: n = 61, > 9 m clemastine: n = 16). Overall, membrane potential tended to become more hyperpolarized with age (p = 0.04; two-way ANOVA, age main effect), in particular 6 m and > 9 m clemastine-treated OPCs were more hyperpolarized than 1 m clemastine-treated OPCs (1 m: − 27.87 ± 3.24 mV, n = 22; 6 m: − 40.32 ± 2.10 mV, n = 61; > 9 m: − 36.76 ± 3.81 mV, n = 16; 1 m vs 6 m: p = 0.01; 6 m vs 9 m p = 0.9; Holm–Bonferroni post-hoc test; Supplementary Fig. S1). Clemastine treatment did not affect membrane capacitance (p = 0.7; two-way ANOVA, condition main effect; 1 m control: n = 17, 6 m control: n = 60, > 9 m control: n = 12, 1 m clemastine: n = 29, 6 m clemastine: n = 77, > 9 m clemastine: n = 23), which decreased after 1 m, as in control animals (Supplementary Fig. S1), as previously reported^13^.

In control animals, neither Na_V_ density nor the proportion of OPCs with Na_V_ differed with age after 1 m (density, p = 0.8; two-way ANOVA, age main effect; 1 m control: n = 46, 6 m control: n = 70, > 9 m control: n = 22, 1 m clemastine: n = 29, 6 m clemastine: n = 77, > 9 m clemastine n = 23; Fig. 1d–f), as previously described^13^, and clemastine treatment did not alter this (p = 0.3; two-way ANOVA, condition main effect). Similarly, in both control and clemastine-treated animals, the proportion of cells with detectable kainate-evoked currents and AMPAR/KAR current density were stable with age (density, p = 0.4; two-way ANOVA, age main effect; 1 m control: n = 15, 6 m control: n = 30, > 9 m control: n = 14, 1 m clemastine: n = 8, 6 m clemastine: n = 37, > 9 m clemastine n = 8; Fig. 1g–i)^13^. However, overall, OPCs in clemastine-treated mice had lower AMPAR/KAR density than in control mice (p = 0.008; two-way ANOVA, condition main effect; Fig. 1h), although the proportion of cells with AMPARs/KARs did not differ between conditions (all ages pooled, control: 96.83%, n = 63; clemastine: 94.74%, n = 57; p = 0.9, χ^2^).

In control animals, NMDAR density decreased between 1 and 6 m (p = 5.5 × 10^–5^; Holm-Bonferroni post-hoc test; 1 m control: n = 20, 6 m control: n = 32; Fig. 1j,k). Although a previous study suggests that NMDAR currents are no longer detected in 6 m cortical OPCs^13^, we found that 22% of OPCs retain NMDARs at this age, nonetheless a significant reduction from the 85% of 1 m OPCs with NMDARs (p = 3.2 × 10^–5^; χ^2^; 1 m: n = 20, 6 m: n = 32). We did not detect NMDA-evoked currents at > 9 m (Fig. 1j–l), consistent with previous reports^13^. Clemastine treatment did not alter the overall NMDAR density (p = 0.07; two-way ANOVA, condition main effect; 1 m control: n = 20, 6 m control: n = 32, 9 m control: n = 10, 1 m clemastine: n = 10, 6 m clemastine: n = 40, 9 m clemastine n = 11), but remarkably, clemastine prevented the decline in NMDARs in 6 m OPCs, with receptor density and the proportion of cells with detectable NMDA-evoked currents similar to that of clemastine-treated 1 m OPCs (p = 0.5, Holm–Bonferroni post-hoc; p = 0.9, χ^2^; respectively; 1 m: n = 10, 6 m: n = 40 Fig. 1j–l). However, clemastine did not revert the loss of NMDARs when given at > 9 m. Taken together, these data indicate that clemastine administration lowers the overall AMPAR/KAR density and extends the window of NMDAR surface expression in middle-aged cortical OPCs, possibly by reverting OPCs into an NMDAR-rich state that is characteristic of postnatal OPCs present at the peak of myelination^13^, although the potency to revert to or maintain an NMDAR-rich state is lost as the animal ages.

### Clemastine does not alter proliferation nor differentiation

AMPAR/KAR signalling modulates OPC proliferation^23–27^, and NMDAR signalling underlies activity-dependent myelination and remyelination^17^. As clemastine reduced AMPAR/KAR density and lengthened the time of NMDAR surface expression, we therefore asked whether it altered cell fate.

**Figure 2:**
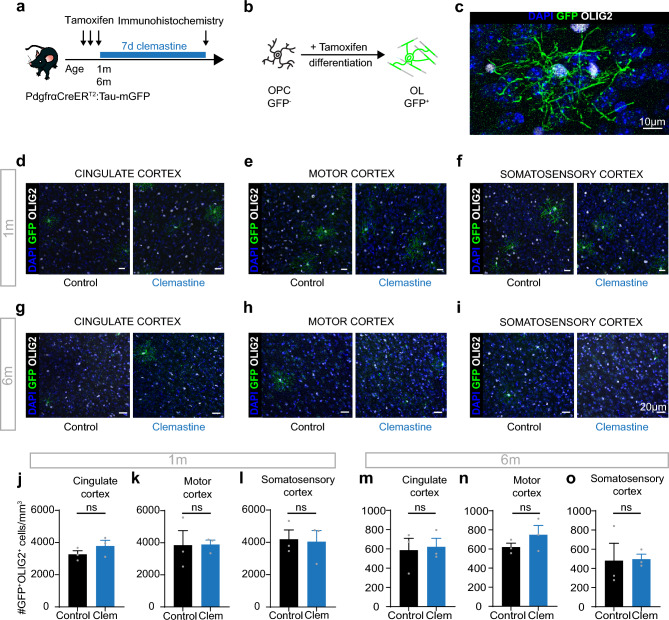
Clemastine does not promote differentiation in homeostatic conditions. (**a**) Experimental design. Following 3 days of tamoxifen administration to induce Cre activity, 1 m or 6 m PdgfrαCreER^T2^:Tau-mGFP mice were given clemastine for 7 days in their drinking water before perfusion-fixation for immunohistochemical analysis. (**b**) Following tamoxifen administration in PdgfrαCreER^T2^:Tau-mGFP mice, newly differentiated oligodendrocytes are mGFP^+^. (**c**) OLIG2^+^mGFP^+^ newly differentiated mature oligodendrocyte. Scale bar 10 µm. (**d–f**) mGFP^+^OLIG2^+^ newly differentiated oligodendrocytes in the cingulate (**d**), motor (**e**) and somatosensory (**f**) cortices in control (left) and clemastine-treated (right) 1 m old mice. Scale bar 20 µm. (**g**–**i**) mGFP^+^OLIG2^+^ newly differentiated oligodendrocytes in the cingulate (**g**), motor (**h**) and somatosensory (**i**) cortices in control (left) and clemastine-treated (right) 6 m old mice. Scale bar 20 µm. (**j**–**o**) Clemastine administration did not increase the number of newly formed oligodendrocytes. Data are shown mean ± SEM, with grey dots indicating individual animals. Statistics were calculated by unpaired two-tailed t-tests, n = 3 for each group.

To examine whether clemastine alters OPC proliferation, we administered clemastine for 7–10 days to 1 m, 6 m, and > 9 m NG2-EYFP mice and labelled proliferating cells with an antibody against the proliferation marker KI67. Clemastine treatment did not alter cortical OPC proliferation at any age (6 m: control, n = 9, clemastine, n = 6; p = 0.6; > 9 m: control, n = 6, clemastine, n = 4; p = 0.5; unpaired two-tailed t-tests; Supplementary Fig. S2). We next asked if clemastine increased differentiation. We first administered clemastine for 7 days to PdgfrαCreER^T2^:Tau-mGFP mice^4^, in which, following tamoxifen administration, newly differentiated oligodendrocytes express membrane-bound GFP (mGFP; Fig. 2a–c). Although clemastine treatment augments the pool of OPCs with NMDA receptors, it did not alter the number of mGFP^+^ cells in the cingulate, motor, or somatosensory cortices of either 1 m or 6 m old mice (1 m: cingulate, p = 0.3, motor, p = 0.97, somatosensory, p = 0.9; 6 m: cingulate, p = 0.8, motor, p = 0.3, somatosensory, p = 0.9; unpaired two-tailed t-tests; n = 3 for all conditions; Fig. 2d–o), nor the overall number of oligodendrocyte lineage cells (p = 0.6; unpaired two-tailed t-test; n = 3 for all conditions; Supplementary Fig. S3).

To test whether allowing a longer time period for differentiation to occur in 6 m animals^28^, or a longer administration period^6^ would reveal an effect on differentiation, we gave 6 m PdgfrαCreER^T2^:Tau-mGFP mice clemastine for 7 or 21 days and measured the number of newly formed mGFP^+^ oligodendrocytes on day 21 (Supplementary Fig. S3), or gave them clemastine for 21 days and measured differentiation on day 35 (Supplementary Fig. S3). These protocols did not result in increased differentiation in the cingulate cortex (p = 0.054, one-way ANOVA, control: n = 9, 7 days clemastine: n = 4, 21 days clemastine: n = 6; p = 0.8, unpaired two-tailed t-test, control: n = 4, clemastine: n = 4; respectively). Finally, we tested whether administering a higher clemastine concentration (see “Methods”) would promote differentiation (Supplementary Fig. S3). Increasing the clemastine concentration did not alter the number of newly differentiated oligodendrocytes in the cingulate cortex (control: 985.1 ± 105.3 cells/mm^3^, n = 9; clemastine: 612.5 ± 137.3 cells/mm^3^, n = 4; p = 0.07; unpaired two-tailed t-test). These findings indicate that although clemastine modulates glutamate receptors in OPCs, this alone is not sufficient to promote differentiation and may suggest that in homeostatic conditions other differentiation cues like those present in a remyelinating lesion or a learning paradigm, such as alteration in neuronal activity, are needed. Our data indicate that clemastine modulates glutamate receptors in OPCs, making OPCs more sensitive to neuronal activity, although clemastine alone is not sufficient to extend the window of NMDAR surface expression by more than a few months and thus, is unlikely to overcome the age-related block of OPC differentiation.

### Metformin extends the window of NMDAR surface expression in aged mice

**Figure 3:**
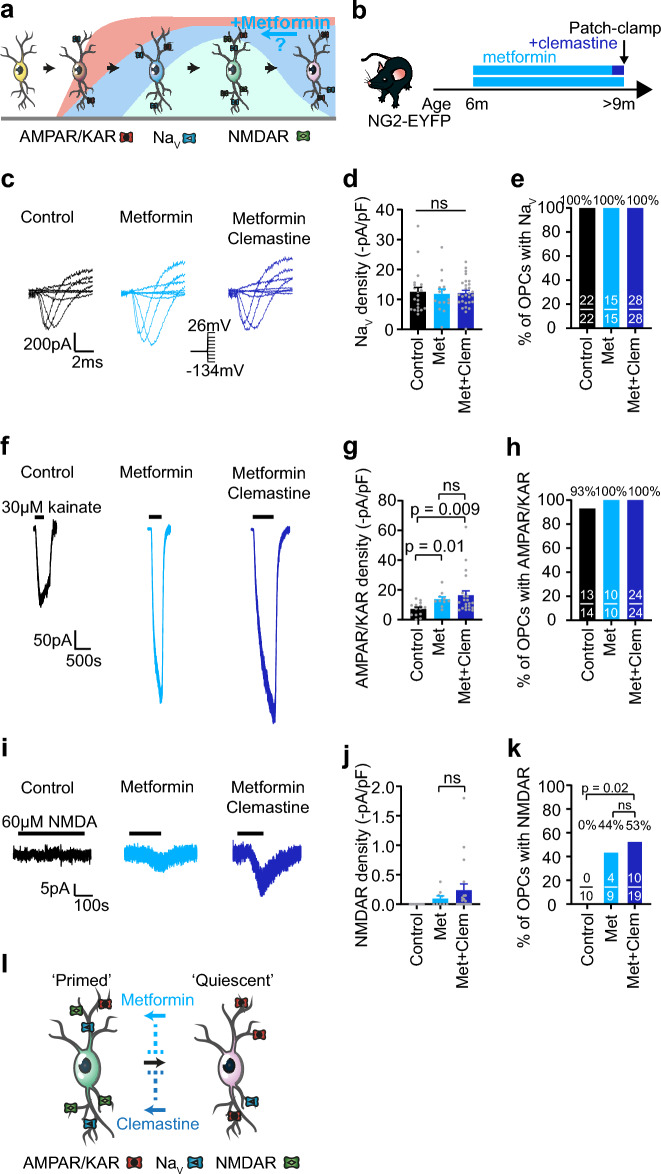
Metformin administration prevents loss of NMDA receptors at 9 months. (**a**) OPCs acquire different voltage-gated ion channels and glutamate receptors with age, with at least five different electrophysiological profiles identified by Spitzer et al.^13^. The curves represent the proportion of OPCs with AMPARs/KARs (red), Na_V_ (blue), and NMDARs (green) with age. Adapted from Spitzer et al., Neuron 2019^13^ with permission, CC BY. (**b**) 6 m mice were given metformin in their drinking water for 3 months before patch-clamp recordings. A second group of mice was given clemastine in addition to metformin during the last 7–10 days before patch-clamp recordings. (**c**) Leak-subtracted traces of Na_V_ currents in response to 20 mV steps from a holding potential of − 74 mV (inset, voltage steps from − 134 to + 26 mV) in cortical OPCs from control (black), metformin (light blue) and metformin + clemastine-treated (dark blue) > 9 m mice. (**d**) Metformin or metformin + clemastine treatment do not alter Na_V_ density (p = 0.9, one-way ANOVA, control: n = 22, metformin: n = 15, metformin + clemastine: n = 28). (**e**) The proportion of OPCs with Na_V_ was unaltered by metformin or metformin + clemastine treatment. (**f**) 30 µM kainate-evoked currents in cortical OPCs from control (black; also shown in Fig. 1g), metformin (light blue) and metformin + clemastine-treated (dark blue) > 9 m mice. (**g**) Metformin and metformin + clemastine administration increased AMPAR/KAR density in OPCs (p = 0.04, one-way ANOVA, control: n = 14, metformin: n = 10, metformin + clemastine: n = 22), but did not differ from each other. P values on the bar graph are from Holm–Bonferroni post-hoc tests. (**h**) The proportion of cells with AMPARs/KARs did not differ with metformin treatment. (**i**) 60 µM NMDA-evoked currents in cortical OPCs from control (black; also shown in Fig. 1j), metformin (light blue) and metformin + clemastine-treated (dark blue) > 9 m mice. Metformin and metformin + clemastine treatments lead to detectable NMDA currents in OPCs, in contrast to controls. (**j**) NMDA-evoked current density did not differ between metformin and metformin + clemastine treated-mice (p = 0.2, unpaired two-tailed t-test, metformin: n = 9, metformin + clemastine: n = 19). (**k**) Metformin and metformin + clemastine treatments increased the proportion of OPCs with detectable NMDAR currents from 0% in controls to 44% and 53% in metformin and metformin + clemastine, respectively. P values on the graph are from χ^2^ tests. (**l**) Clemastine and metformin extend the window of NMDAR surface expression by either reversing NMDAR loss or preventing NMDAR downregulation, likely reversing (full arrows) or preventing (dashed lines) the transition from an NMDAR-rich state, perhaps primed to differentiate^13,16^, to a quiescent state, lacking NMDAR receptors. Data are shown as mean ± SEM, with grey dots indicating individual recorded cells in (**d**,**g**,**j**). The numbers on the bars in (**e**,**h**,**k**) indicate the number of responding cells over the number of recorded cells.

As clemastine treatment did not reverse the age-driven loss of NMDARs at > 9 m, we sought to identify a compound that might do so. Metformin, a widely-prescribed drug for type 2 diabetes, was recently shown to promote remyelination in aged rats and to restore OPCs’ responsiveness to pro-differentiation cues, including muscarinic antagonists, in aged OPCs^10^. We therefore asked whether metformin administration could alter OPC membrane properties and restore NMDARs in > 9 m old mice, promoting a specific OPC state (Fig. 3a), and whether combining metformin and clemastine, a muscarinic antagonist, would lead to a more potent effect.

To address this, we administered 300 mg/kg metformin in the drinking water of NG2-EYFP mice for 3 months^10^, between 6 and > 9 m, and like with clemastine we detected no change in water intake between controls or treatment groups, as has previously been reported^29^. A second group of mice were dosed with 20 mg/L clemastine in addition to metformin during the last 7–10 days before single-whole-cell patch-clamp recordings (Fig. 3b). Metformin treatment alone or in combination with clemastine did not alter most passive membrane properties, i.e. membrane resistance or capacitance (p = 0.1, one-way ANOVA, control: n = 9, metformin, n = 14, metformin + clemastine, n = 27; p = 0.4, one-way ANOVA, control: n = 12, metformin: n = 15, metformin + clemastine, n = 28; respectively), but metformin-treated OPCs had a larger inward rectifying K^+^ conductance (p = 0.004; one-way ANOVA; control: n = 9, metformin, n = 14, metformin + clemastine, n = 27) and were therefore more hyperpolarized (p = 0.007; one-way ANOVA; control: n = 9, metformin, n = 14, metformin + clemastine, n = 27), but these differences did not hold in the presence of clemastine (Supplementary Fig. S4).

Na_V_ density and the proportion of cells with Na_V_ were unaltered by metformin administration (density: p = 0.9; one-way ANOVA; control: n = 22, metformin: n = 15, metformin + clemastine: n = 28; and 100% of recorded OPCs had Na_V_ currents in all conditions; Fig. 3c–e). In contrast, AMPAR/KAR density increased with metformin treatment, (p = 0.04; one-way ANOVA; control: n = 14, metformin: n = 10, metformin + clemastine: n = 22; Fig. 3f–h); the addition of clemastine did not potentiate this effect (p = 0.4; Holm–Bonferroni post-hoc; metformin: n = 10; metformin + clemastine: n = 22). Remarkably, we detected NMDA-evoked currents in 44% of metformin-treated OPCs, at an age when NMDA did not evoke any detectable currents in OPCs in control animals (Fig. 3i–k). The proportion of NMDA-responsive OPCs in metformin-treated mice did not differ from that in 1 m control mice (p = 0.07; χ^2^; 1 m control: n = 20, 9 m metformin: n = 9). However, the NMDAR densities in 9 m metformin-treated mice (0.1 ± 0.04 pA/pF; n = 9) were similar to NMDAR densities detected in 6 m control mice (0.04 ± 0.02 pA/pF; n = 32; p = 0.2, unpaired two-tailed t-test), but lower than NMDAR densities in control 1 m mice (0.39 ± 0.06 pA/pF; n = 20; p = 0.0008, unpaired two-tailed t-test), indicating that metformin increases the proportion of OPCs with NMDARs, but does not potentiate NMDA-evoked current size. These data suggest that metformin can revert and/or prevent the loss of NMDARs in > 9 m mice. Additional treatment with clemastine did not enhance this effect further (density, p = 0.2, unpaired two-tailed t-test; proportion, p = 0.7, χ^2^; metformin: n = 9, metformin + clemastine: n = 19; Fig. 3j,k).

Taken together, our data indicate that metformin administration can prevent or even revert NMDAR downregulation in cortical OPCs when administered between 6 and > 9 m, and suggest that like clemastine, metformin promotes an NMDAR-rich OPC state (Fig. 3l).

### Metformin promotes differentiation in aged mice

**Figure 4:**
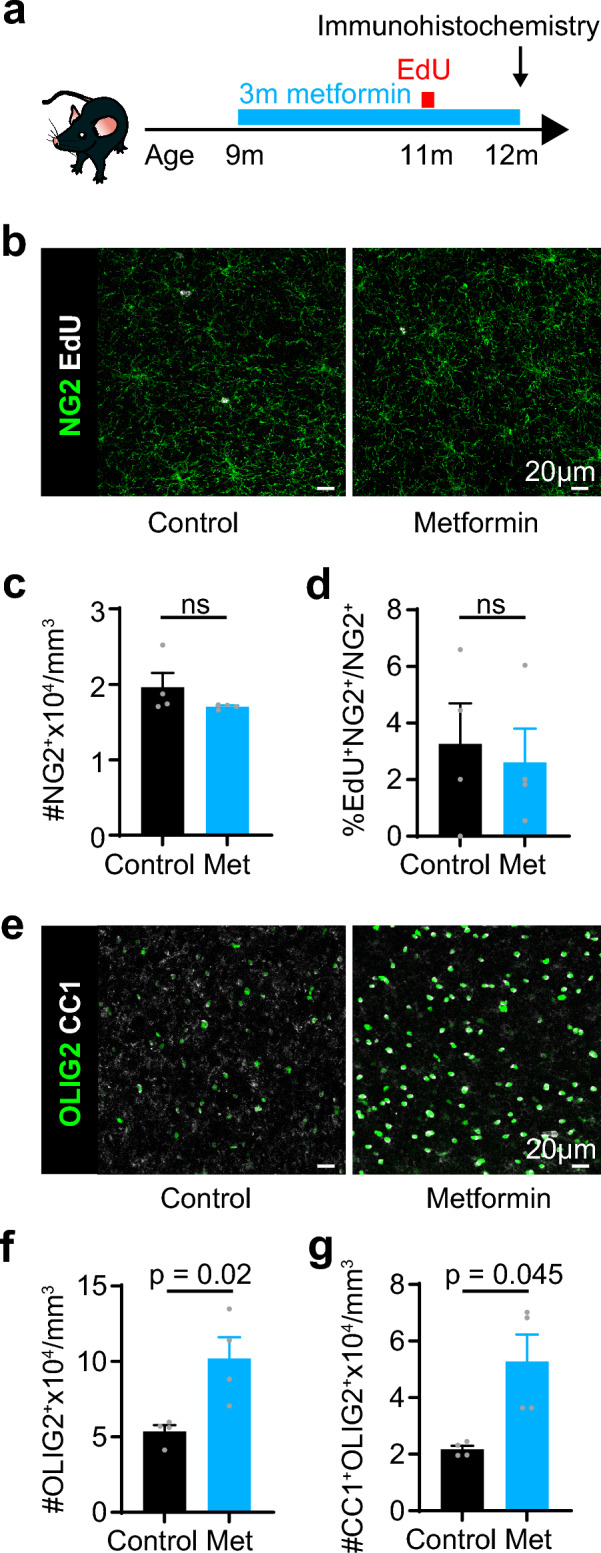
Metformin administration promotes differentiation. (**a**) Experimental design. 9 m mice were given metformin in their drinking water for 3 months before perfusion-fixation for immunohistochemical analysis. After 2 months, EdU was added to the drinking water for 96 h. (**b**) The cingulate cortex was immunolabelled against NG2 and EdU to assess proliferation. Scale bar 20 µm. (**c**) The density of OPCs (NG2^+^ cells) did not differ with metformin administration. (**d**) The proportion of OPCs cells that incorporated EdU did not differ with metformin administration. (**e**) The cingulate cortex was immunolabelled against OLIG2 and CC1 to assess differentiation. Scale bar 20 µm. (**f**) The density of oligodendrocyte lineage cells (OLIG2^+^ cells) increased with metformin treatment. (**g**) Metformin increased the density of oligodendrocytes (CC1^+^OLIG2^+^ cells). Data are shown as mean ± SEM, with grey dots indicating individual animals. Statistics were calculated by unpaired two-tailed t-tests, n = 4 for each group.

As both clemastine and metformin extended the window of NMDAR surface expression but clemastine did not modulate cell fate in OPCs, we next asked whether metformin treatment could alter OPC fate. To test this, we administered 300 mg/kg metformin in the drinking water of mice for 3 months, between 9 and 12 m. After 2 months, we administered 0.2 mg/mL EdU (5-ethynyl 2′-deoxyuridine) for 96 h to label dividing cells (Fig. 4a). Although metformin treatment did not alter OPC density or proliferation in the cingulate cortex (Fig. 4b–d), it increased the density of mature oligodendrocytes (control: 2.17 × 10^4^ ± 1.25 × 10^3^ cells/mm^3^; metformin: 5.28 × 10^4^ ± 9.49 × 10^3^ cells/mm^3^; p = 0.045; unpaired two-tailed t-test; n = 4 in both conditions), resulting in an increase in oligodendrocyte lineage cell density (control: 5.36 × 10^4^ ± 4.18 × 10^3^ cells/mm^3^; metformin: 10.19 × 10^4^ ± 1.41 × 10^4^ cells/mm^3^; p = 0.02; unpaired two-tailed t-test; n = 4 in both conditions; Fig. 4e–g). This suggests that metformin treatment may promote differentiation, rather than proliferation, in aged animals. While we cannot exclude that this increase in mature oligodendrocytes results from enhanced survival of existing cells, we measured oligodendrocytes at 12 m, when the number of oligodendrocytes in the cortex is still increasing^5^ and there is no detectable loss of myelinating oligodendrocytes^30,31^, suggesting that the increase we observed is primarily from enhanced differentiation.

Taken together, our data show that the pro-remyelination compounds clemastine and metformin modulate OPC membrane properties, lengthening the window of NMDAR surface expression. Metformin extended the age at which OPCs with NMDARs were detectable even further than clemastine did, and, in contrast to clemastine, promoted differentiation in aged animals, suggesting that metformin is a more potent modulator than clemastine.

## Discussion

We sought to determine whether clemastine and metformin enhance remyelination by altering OPC membrane properties and promoting a specific OPC state. We recently proposed that the different electrophysiological profiles we detected in OPCs could represent at least five functional cell states: naïve, lacking ion channels and glutamate receptors; migratory; highly proliferative; primed for differentiation, marked by NMDAR surface expression; and quiescent, marked by the loss of NMDARs and increased AMPAR/KAR current density^13,16^. We found that both clemastine and metformin modulate these ion channels and glutamate receptors in OPCs, and extend the window of NMDAR surface expression in ageing mice, perhaps preventing or reversing the transition from a state primed for differentiation to a quiescent state (Fig. 3l), in line with a report suggesting that clemastine prevents the accumulation of senescent OPCs^32^. As OPC NMDARs have been shown to play a role in remyelination^17^, maintaining OPCs in an NMDAR-rich state may partially underlie the pro-remyelination properties of clemastine and metformin.

Although clemastine maintained a higher proportion of OPCs with NMDARs at 6 m, it did not have any clear effect on OPC fate, in contrast to reports suggesting that clemastine promotes OPC differentiation in vivo. However, we administered clemastine in the absence of any other experimental paradigm, whereas in other studies, clemastine is administered during or after hypoxic injury^33^, prior to a demyelinating injury^9^ or prior to a learning task^6^. It is conceivable that while clemastine alters OPCs’ sensitivity to neuronal activity, further cues such as those present following injury or learning may be required to promote differentiation. In line with this, a study reports that clemastine promotes remyelination and functional recovery following cuprizone-induced demyelination, but has no effect on myelination when given to control animals^34^. This type of attribute is ideal for a candidate drug to promote myelin regeneration, as it would be unlikely to promote ectopic differentiation and myelination outside of a lesion.

In addition to clemastine extending the window of NMDAR surface expression to 6 m, there may be a trend for clemastine to reduce NMDAR density at 1 m (Fig. 1). Along with a reduction in overall AMPAR/KAR density with clemastine treatment, this suggests that clemastine might push OPCs back to a P0-like phenotype^13^ when administered at 1 m. This may point to a general effect of clemastine preventing or reverting age-driven state transitions, and would be interesting to explore further at a later stage. Nonetheless, as NMDARs contribute to remyelination^17^ and loss of NMDARs correlates with the decline in remyelination efficacy^13^, focusing on preventing their loss in ageing as we have shown here with both clemastine and metformin may be a valuable therapeutic strategy.

Nevertheless, clemastine did not prevent the loss of NMDARs at 9 m. Recent studies in rodents have demonstrated that timing of intervention is critical to promote remyelination^35^, and that pro-differentiation compounds show reduced efficacy with age^10^. In addition, multiple sclerosis clinical trial data suggest that some compounds may show higher efficacy to promote remyelination in younger compared to older patients^36^. In line with this, our findings highlight that clemastine may be useful to promote remyelination in younger patients, but perhaps not in middle-aged patients and above.

In contrast to clemastine, metformin both increased the proportion of OPCs with NMDARs at 9 m and promoted differentiation in aged mice, in line with a study suggesting that metformin promotes remyelination in aged rats^10^, although it is important to note our data do not reveal whether the increase in differentiation we detected resulted in increased myelination. Surprisingly, metformin also increased AMPAR/KAR density, perhaps underlying its enhanced efficacy compared to clemastine. However, this appears at odds with the “rejuvenating” effect of metformin, as in controls AMPAR/KAR density increases in the cortex by the third postnatal week^13^ to reach a plateau (Fig. 1) and may be associated with reduced proliferation and differentiation^13,16,26^. Metformin is thought to promote OPC differentiation in vitro or following demyelination through activation of AMPK signalling^10,37,38^, which has been shown to regulate ion channels and glutamate receptors in neurons and Müller cells^39,40^. Thus, it is conceivable that metformin treatment modulates OPC membrane properties and promotes differentiation in aged mice through activation of AMPK.

Both metformin and clemastine have been shown to act on other CNS cells such as microglia^41–43^, and it is therefore possible that metformin and clemastine modulate OPC membrane properties indirectly. For instance, cytokine signalling from glial cells can modulate glutamate receptors in neurons^44^, and inflammatory cytokines are known to be increased in the ageing brain^45,46^, while both clemastine and metformin have been shown to reduce pro-inflammatory cytokine release as well as microglia and astrocytic reactivity^41,47^. One further intriguing possibility is that both metformin and clemastine have been shown to promote overall brain-derived neurotrophic factor (BDNF) expression^37,48^, which increases NMDAR currents in OPCs^17^, but is reduced with age in the cortex^49^. However, knocking out the M1 muscarinic receptor in OPCs recapitulates the effect of clemastine on differentiation, suggesting a more direct action^6,50^. As both G protein-coupled signalling and growth factors can modulate ion channels in OPCs^17,20^, it is possible that the combined direct and indirect effects of clemastine extend the window of NMDAR surface expression in OPCs. Nevertheless, as metformin both altered membrane properties, making OPCs more sensitive to differentiation cues like neuronal activity, and promoted differentiation, it is possible that it is either more potent than clemastine, or further acts on the environment in a way that would promote OPC differentiation, perhaps by reducing ageing-induced inflammation^47,51^.

In summary, our findings show that clemastine and metformin can alter OPC membrane properties, reinforcing the idea that OPCs exist in different functional states that can be modulated. Our data provide a potential mechanism for the pro-remyelination properties of metformin and clemastine, and highlight that further understanding OPC functional states and dissecting the mechanisms to induce or prevent state transitions is likely to lead to new therapeutic targets to promote myelin repair.

## Methods

### Animals

Experiments were performed in accordance with EU guidelines for the care and use of laboratory animals, and with the guidelines of the UK Animals (Scientific Procedures) Act 1986 and subsequent amendments. Use of animals in this project was approved by the Animal Welfare and Ethical Review Body for the University of Cambridge and carried out under the terms of UK Home Office Licenses and in line with ARRIVE guidelines. All mice were maintained under a 12 h light:12 h dark cycle with food and water supplied ad libitum. To identify OPCs in acute brain slices, we used heterozygous knock-in NG2-EYFP transgenic mice^22^, kindly donated by Jacqueline Trotter, in which NG2^+^ cells express EYFP. OPCs were recorded in P20–35 (1 m), P180–220 (6 m), or P270–350 (> 9 m) mice. To label newly differentiated oligodendrocytes, we used PdgfraCreER^T2^:Tau-mGFP mice^4^ aged P20–35 (1 m) or P180–220 (6 m). When administering metformin for immunohistochemical analysis, we used 12 m surplus Cre stock animals, as endogenous labelling for specific stages of the oligodendrocyte lineage was not required.

### Acute brain slices

Brain slices were prepared as previously described^13,52^. Briefly, 225 µm-thick coronal slices were cut from NG2-EYFP mice between P20 and P350 in ice-cold (~ 1 °C) oxygenated (5% CO_2_/95% O_2_) bicarbonate-buffered aCSF containing, in mM: 124 NaCl, 26 NaHCO_3_, 1 NaH_2_PO_4_, 2.5 KCl, 2 MgCl_2_, 2.5 CaCl_2_, 10 glucose, pH 7.4, 330 mOsm. 1 mM kynurenic acid was added to block glutamate receptors that might be activated during dissection^52^.

### Electrophysiology

All experiments were performed in whole-cell voltage-clamp mode, with junction potential (− 14 mV) compensated holding potential − 74 mV. Pipette resistance was between 4.4 and 6.5 MΩ and mean uncompensated series resistance was 25 MΩ. Recordings were performed in HEPES-buffered aCSF containing, in mM: 144 NaCl, 10 HEPES, 1 NaH_2_PO_4_, 2.5 KCl, 2.5 CaCl_2_ and 10 glucose, with pH adjusted to 7.2–7.4 with 1 M NaOH, and osmolarity 315 mOsm. Mg^2+^ was omitted to record NMDA-evoked responses. The internal solution contained, in mM: 130 Cs-gluconate or K-gluconate, 4 NaCl, 0.5 CaCl_2_, 10 HEPES, 10 BAPTA, 4 Mg_x_ATP, 0.5 Na_x_GTP, and 2K-Lucifer Yellow, pH adjusted to 7.2–7.4 with 2M CsOH or KOH, with osmolarity between 290 and 300 mOsm. All recordings took place at room temperature (21 °C), and the recording solution was continuously oxygenated with 100% O_2_. Inclusion criteria was based on series resistance, leak current being lower than 400 pA and a stable baseline. An Axopatch 200 (Molecular Devices) was used for voltage-clamp data acquisition. Voltage step data were sampled at 50 kHz and filtered at 10 kHz and drug application data were sampled at 1 kHz using pClamp 10.7 or pClamp 11.2 (Molecular Devices). Cells were recorded in cortical layers 2–6. During recordings, cells were filled with Lucifer Yellow. Location and cell identity were confirmed by post-hoc immunohistochemistry against GFP and OLIG2. In 15/15 cases, imaged cells were positive for EYFP and OLIG2.

### Drugs

100 µM glycine, an NMDA co-agonist, was included in the recording solution. 5 µM strychnine, a glycine receptor antagonist, was also included to avoid activation of glycine receptors. 60 µM NMDA was used to activate NMDARs, and 30 µM kainate was used to activate both AMPARs and KARs. 200 µM BaCl_2_ was added to the recording solution after determination of passive membrane properties to block inward rectifying K^+^ currents^13,52^.

### Electrophysiological analysis

Series resistance, membrane resistance, and membrane capacitance were calculated as previously described^13,52,53^ using a custom written MATLAB script. Resting membrane potential and the slope of the inward rectifying K^+^ current (inward conductance; measured as the highest slope with the best linear fit between − 34 and − 134 mV) were manually calculated in Excel.

### Clemastine administration

20 mg/L clemastine fumarate (Sigma-Aldrich; SML0445) was administered in drinking water for 7–10 days for patch-clamp experiments. For immunohistochemistry experiments, clemastine was administered for 7 or 21 days. The drinking water concentration was determined by calculating an equivalent concentration to 10 mg/kg^9,54^ taking into account an average C57BL/6 mouse weight of 12 g (at P25) and an average water consumption of 6 mL/day. The drinking water concentration (20 mg/L) was then kept constant at different ages, as water intake is positively correlated with body weight^55^. In one instance, as specified in the results section, the equivalent concentration to 10 mg/kg was recalculated in 6 m mice after their water consumption was found to be lower than expected (taking into account an average weight of 33.1 g and an average water consumption of 4.2 mL/day, the drinking water concentration was 79 mg/L). Clemastine administration did not alter water consumption levels.

### Metformin administration

2 mg/mL metformin hydrochloride (ApexBio; B1970) was administered in drinking water for 3 months. Mice were weighed weekly, and the drinking water concentration was determined by calculating an equivalent concentration to 300 mg/kg^10^ based on an average drinking water consumption of 15 mL/100g^56^. Metformin administration did not alter water consumption levels.

### EdU administration

0.2 mg/mL EdU (Life Technologies, E10187)^4^ was administered in drinking water for 96 h. EdU administration did not alter water consumption levels.

### Induction of Cre-mediated recombination

PdgfrαCreER^T2^:Tau-mGFP mice were given daily doses of tamoxifen by oral gavage for three consecutive days to induce Cre activity. 1 m mice were given 150 mg/kg tamoxifen, and 6 m animals were given 300 mg/kg tamoxifen.

### Immunohistochemistry and imaging

Immunohistochemistry was performed as previously described^52,57^. Mice were perfused-fixed with ice-cold PBS followed by ice-cold 4% PFA. Dissected brains were postfixed overnight at 4 °C or for one hour at 21 °C for NG2 labelling before being washed in PBS. 100 µm-thick slices were cut on a vibrating microtome. Alternatively, acute brain slices were incubated in 4% PFA for one hour at room temperature, before being washed in PBS. For antibody labelling, free-floating slices were incubated in 10% goat serum and 0.5% Triton-X 100 in PBS for 4–5 h at room temperature, on a rotating shaker. Slices were incubated with primary antibodies in PBS overnight at room temperature. Primary antibodies were as follows: chicken anti-GFP, 1:1000 (Abcam, ab13970); rabbit anti-OLIG2, 1:300 (EMD Millipore, AB9610); rabbit anti-KI67, 1:300 (Abcam, ab16667); rabbit anti-NG2, 1:300 (Millipore, AB5320); mouse anti-APC, clone CC1, 1:300 (Millipore, MABC200 or OP80). Following three 30 min washes in PBS, the slices were incubated in secondary antibodies in PBS at a 1:1000 concentration overnight at 4 °C or for 5 h at room temperature, on a rotating shaker. Secondary antibodies were as follows: goat anti-chicken IgY Alexa Fluor 448 (Abcam, ab150169), goat anti-chicken IgY Alexa Fluor 568 (Invitrogen, A-11041), goat anti-rabbit IgG Alexa Fluor 488 (Invitrogen, A-11008), goat anti-rabbit IgG Alexa Fluor 568 (Invitrogen A-11036), goat anti-rabbit IgG Alexa Fluor 647 (Invitrogen, A-21245), and goat anti-mouse IgG Alexa Fluor 750 (Invitrogen, A-21037). After two washes in PBS, slices were incubated with 1 ng/mL DAPI for 20 min, and following a final PBS wash, mounted on glass slides.

To visualise the EdU-labelled cells, we used a Click-iT Cell Reaction Buffer Kit (Invitrogen, C10269). Following antibody staining, and immediately after DAPI incubation, slices were washed in 2% BSA for 10 min, then incubated for 30 min with the Click It reaction mix (prepared according to the manufacturer’s instructions; we included an Alexa Fluor 555-conjugated Azide (Invitrogen, A20012) or Alexa Fluor 647-conjugated Azide (Invitrogen A10277)). Following this, slices were washed for 10 min with 2% BSA, and then for 30 min with PBS before mounting them on glass slides.

Samples were imaged on a Leica TCS SP5 microscope or a Leica TCS SP8 microscope. Laser intensity, voltage and offset were adjusted to maximise the signal to noise ratio. Parameters were kept constant for negative control slices. Images were acquired at 600 Hz and frame averaged 2–4 times, as needed. Images of patched cells were obtained with a 63× oil objective. Z-stack thickness depended on cell morphology, and was between 5 and 20 µm. Optical slice thickness was 0.5 µm. Images were visualised and processed in LAS X and FIJI. When imaging to quantify OPC proliferation or differentiation, a 20× objective was used. Z-stack thickness was determined based on OLIG2 signal (when imaging differentiation) or NG2 signal (when imaging proliferation) and was typically 25–35 µm. Optical slice thickness was 1.6 µm. Images were analysed in Fiji and cells were counted manually. Four non-overlapping images were acquired for each region of interest.

### Quantification and statistical analysis

Data are shown as mean ± SEM. Statistics were computed in GraphPad Prism or manually in Excel. When comparing two conditions, unpaired two-tailed t-tests were used; variance was tested by F-test, and Welch’s correction applied if unequal. Proportions were tested with a χ^2^ test, with Yates’ correction for small numbers. When comparing three or more conditions, one-way ANOVA was used; variance was tested with a Brown–Forsythe test, and Welch’s correction was applied if variance was unequal. Post-hoc comparisons were performed with Holm–Bonferroni tests. When comparing age-driven changes in control compared to clemastine-treated animals, a two-way ANOVA was used, followed by Holm–Bonferroni post-hoc tests. For electrophysiological experiments, individual cells were n = 1. For immunohistochemical analyses, individual animals were n = 1. The threshold for statistical significance was p ≤ 0.05.

### Supplementary Information

Supplementary Information.

## Data Availability

All data generated or analysed during this study are included in this published article (and its Supplementary Information files).
