# Supplementary material for: Clemastine and metformin extend the window of NMDA receptor surface expression in ageing oligodendrocyte precursor cells
Source: Sci Rep. 2024 Feb 19;14:4091. doi: 10.1038/s41598-024-53615-x (PMC10876931; doi:10.1038/s41598-024-53615-x)
Supplement: Supplementary file 1 — Supplementary Information. [file 41598_2024_53615_MOESM1_ESM.pdf]

Clemastine and metformin extend the window of NMDA receptor surface expression in ageing oligodendrocyte precursor cells

Yasmine Kamen[1*], Kimberley Anne Evans[1], Sergey Sitnikov[1], Sonia Olivia Spitzer[1], Omar de Faria Jr.[1], Mert Yucel[1], and Ragnhildur Thóra Káradóttir[1,2*]

1. Wellcome – Medical Research Council Cambridge Stem Cell Institute & Department of Veterinary Medicine, University of Cambridge, Cambridge, United Kingdom

2. Department of Physiology, BioMedical Center, Faculty of Medicine, University of Iceland, Reykjavik, Iceland

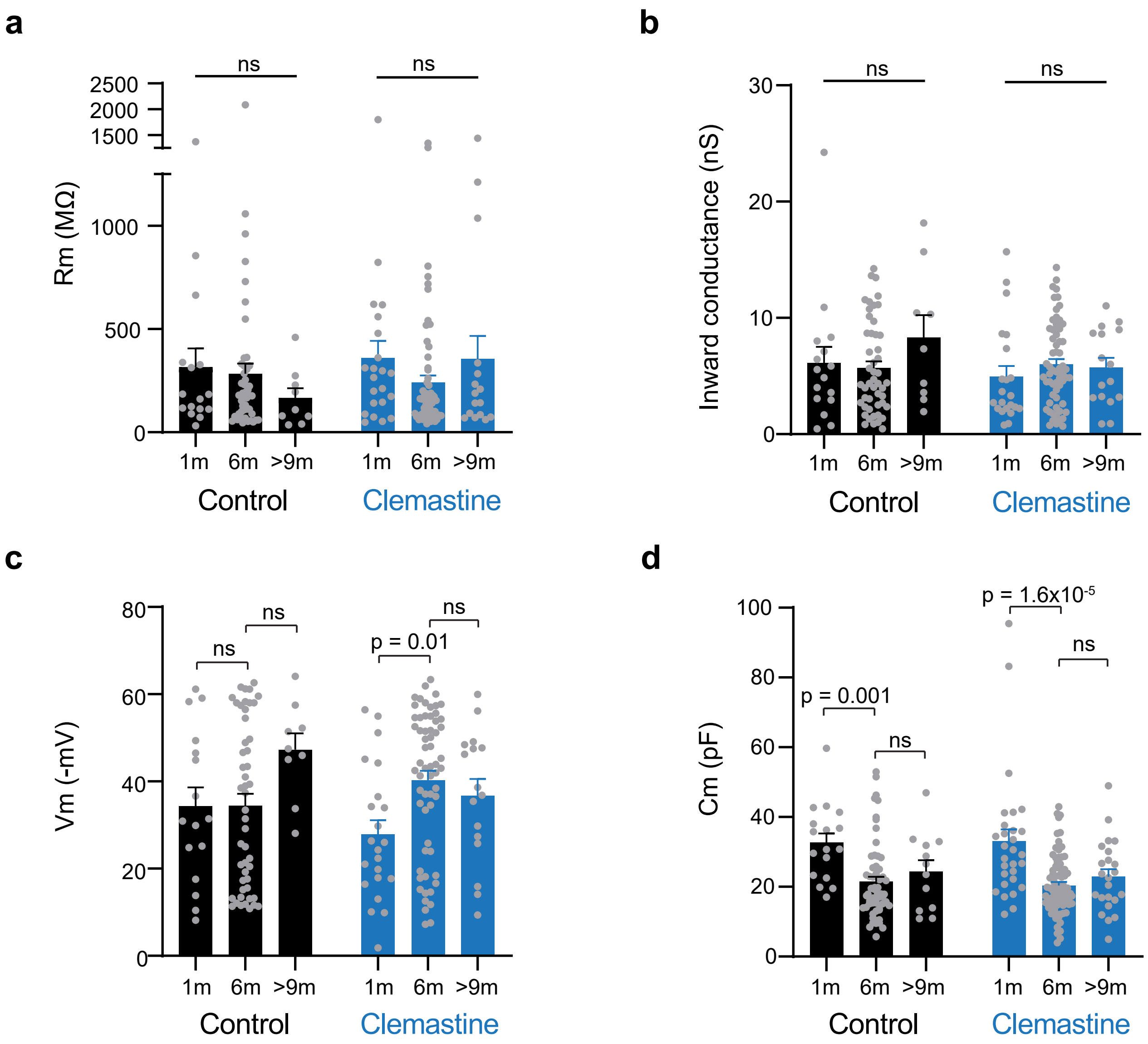

Supplementary Figure S1. Clemastine does not alter OPC passive membrane properties
a Membrane resistance (Rm) does not differ with age or clemastine treatment in adult cortical OPCs. A two-way ANOVA was run to compare control and clemastine-treated OPCs across ages: condition main effect, p=0.3; age main effect, p=0.5; interaction, p=0.3. 1m control: n=16, 6m control: n=49, 9m control: n=9, 1m clemastine: n=22, 6m clemastine:n=61, 9m clemastine: n=16. p-values on the bar graph are from the condition and age main effects. **b** Inward rectifying K^+ conductance (inward conductance) does not differ with age or clemastine treatment in adult cortical OPCs. A two-way ANOVA was run to compare control and clemastine-treated OPCs across ages: condition main effect, p=0.1; age main effect, p=0.4; interaction, p=0.3; 1m control: n=16, 6m control: n=49, 9m control: n=9; 1m clemastine: n=22, 6m clemastine:n=61; 9m clemastine: n=16. p-values on the bar graphs are from condition and age main effects. **c** Resting membrane potential (Vm) was more negative in 6m clemastine-treated mice. In contrast, in control mice, Vm did not change with age. A two-way ANOVA was run to compare control and clemastine-treated OPCs across ages: condition main effect, p=0.2; age main effect, p=0.04; interaction, p=0.04, indicating no difference in Vm between conditions, but a difference in how Vm changes with age with clemastine treatment. 1m control: n=16, 6m control: n=49, 9m control: n=9; 1m clemastine: n=22, 6m clemastine:n=61; 9m clemastine: n=16. p-values on the bar graph are from Holm-Bonferroni post-hoc tests. **d** Membrane capacitance (Cm), a proxy for cell surface area, decreases with age in adult cortical OPCs in both control and clemastine-treated mice. A two-way ANOVA was run to compare control and clemastine-treated OPCs across ages: condition main effect, p=0.7; age main effect, $p=6.5 \times 10^{-8}$; interaction, p=0.9. 1m control: n=17, 6m control: n=60, 9m control: n=12; 1m clemastine: n=29, 6m clemastine:n=77; 9m clemastine: n=23. p-values on the bar graph are from Holm-Bonferroni post-hoc tests. Data are shown as mean±SEM, with grey dots indicating individual recorded cells.

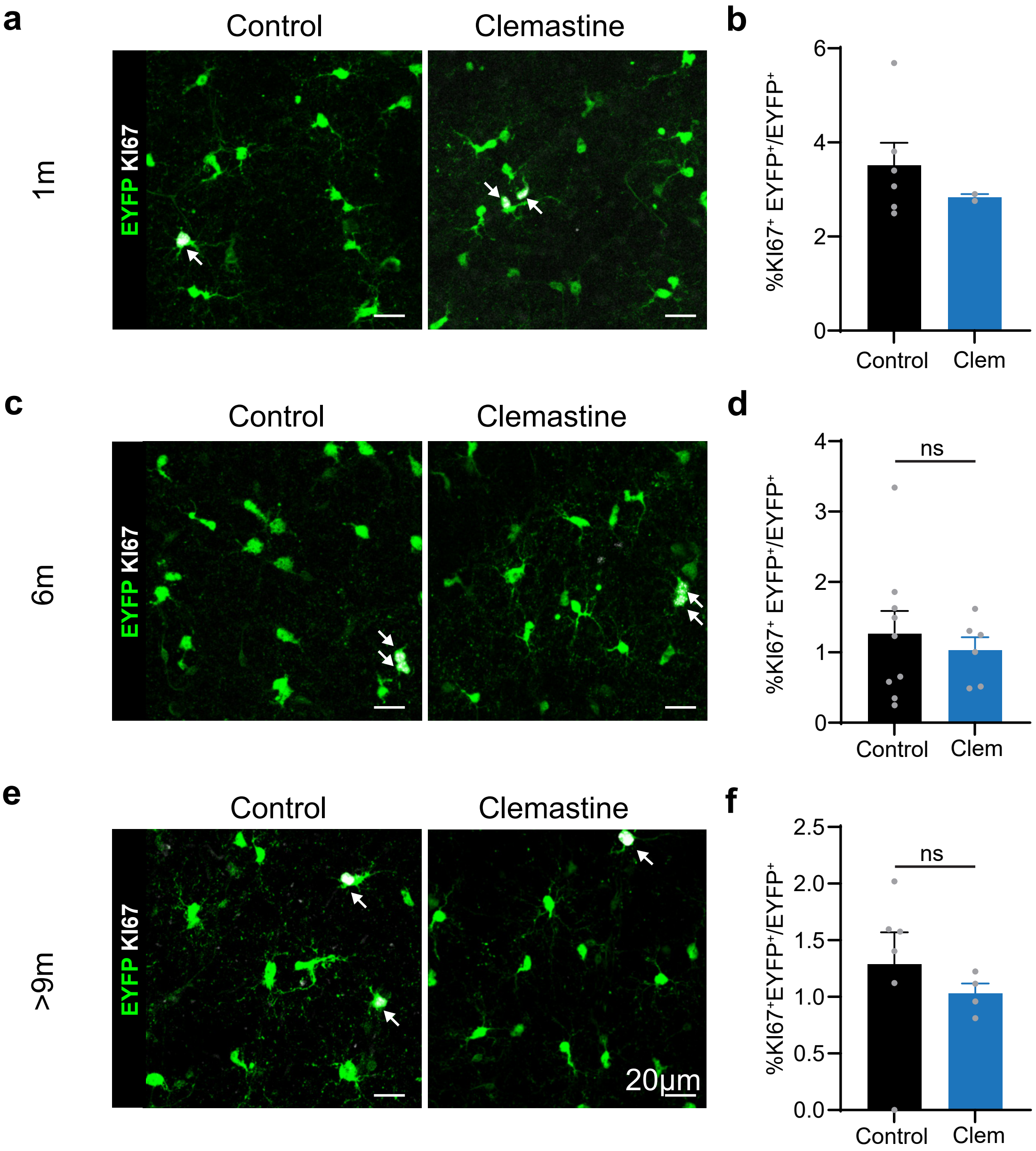

Supplementary Figure S2 Clemastine does not alter OPC proliferation
a Cortical slices from 1m NG2-EYFP mice were immulabelled against EYFP and KI67, a proliferation marker. $EYFP^{+}KI67^{+}$ cells are indicated with white arrows. **b** 7-10 days clemastine treatment is unlikely to alter OPC proliferation in 1m mice, but this was not tested statistically due to a small sample size: control: n=6; clemastine: n=2. **c** Cortical slices from 6m NG2-EYFP mice were immulabelled against EYFP and KI67. $EYFP^{+}KI67^{+}$ cells are indicated with white arrows. **d** 7-10 days clemastine treatment did not alter OPC proliferation in 6m mice. p=0.6, unpaired two-tailed t-test; control: n=9, clemastine: n=6. **e** Cortical slices from >9m NG2-EYFP mice were immulabelled against EYFP and KI67. $EYFP^{+}KI67^{+}$ cells are indicated with white arrows. **f** 7-10 days clemastine treatment did not alter OPC proliferation in >9m mice. p=0.5, unpaired two-tailed t-test; control: n=6, clemastine: n=4. Data are shown as mean ±SEM, with grey dots indicating individual animals.

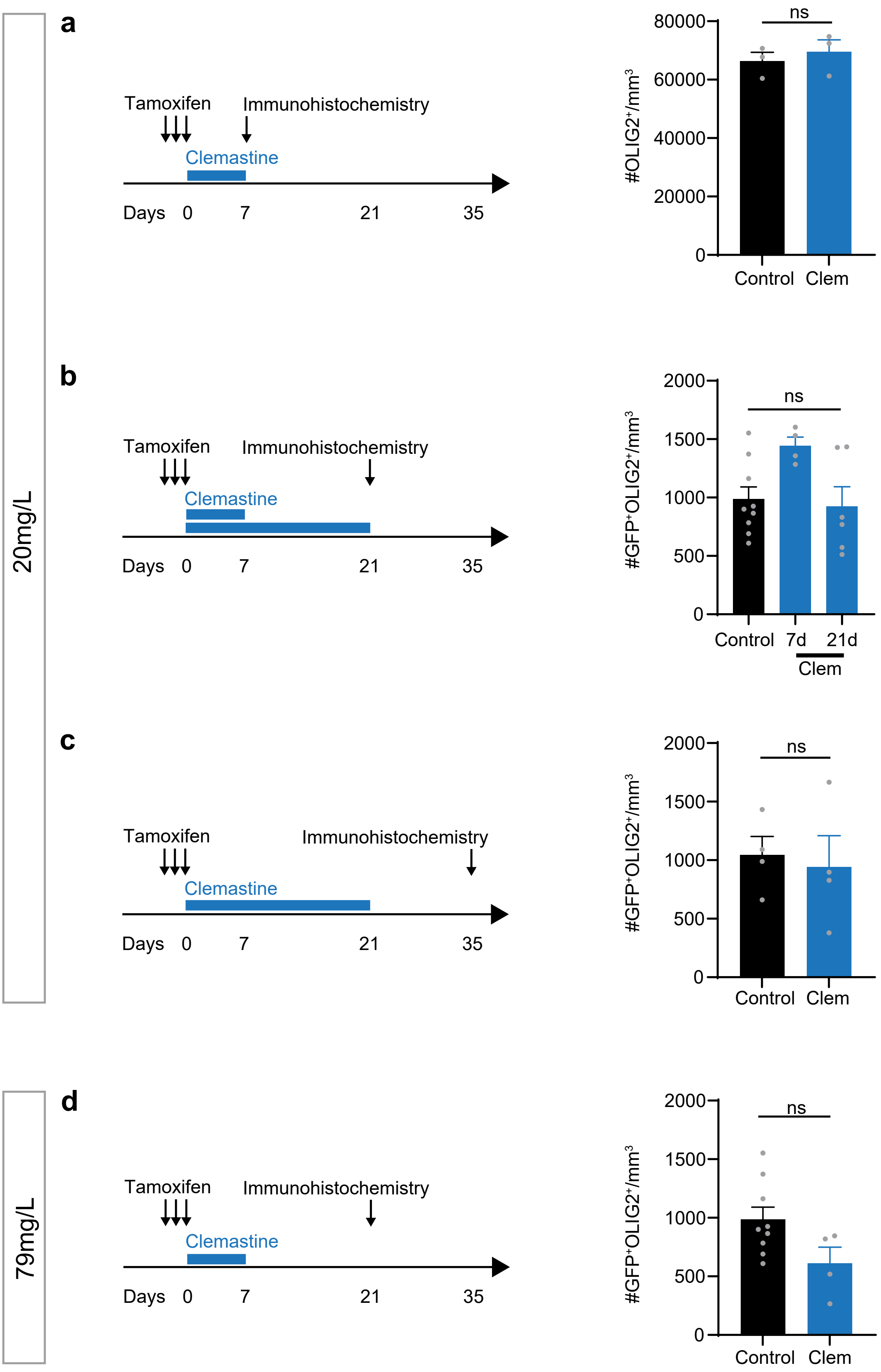

Supplementary Figure S3. Different clemastine administration protocols and doses do not promote differentiation

Supplementary Figure S3. Different clemastine administration protocols and doses do not promote differentiation

a Following 3 days of tamoxifen administration to induce Cre activity, 6m PdgfrαCreER^{T2}:Tau-mGFP mice were given 20mg/L clemastine for 7 days in their drinking water before perfusion-fixation for immunohistochemical analysis. 7 days clemastine treatment did not alter the number of oligodendrocyte lineage cells (OLIG2^{+}). p=0.6, unpaired two-tailed t-test; n=3 for both conditions. **b** Following 3 days of tamoxifen administration to induce Cre activity, 6m PdgfrαCreER^{T2}:Tau-mGFP mice were given 20mg/L clemastine for 7 or 21 days in their drinking water. All mice were perfused-fixed after 21 days for immunohistochemical analysis. Clemastine treatment did not alter the number of newly differentiated oligodendrocytes in the cingulate cortex. p=0.054, one-way ANOVA, control: n=9, 7 days clemastine: n=4, 21 days clemastine: n=6. **c** Following 3 days of tamoxifen administration to induce Cre activity, 6m PdgfrαCreER^{T2}:Tau-mGFP mice were given 20mg/L clemastine for 21 days in their drinking water before perfusion-fixation at 35 days for immunohistochemical analysis. This longer treatment did not alter differentiation in the cingulate cortex. p=0.8, unpaired two-tailed t-test; n=4 for both conditions. **d** Following 3 days of tamoxifen administration to induce Cre activity, 6m PdgfrαCreER^{T2}:Tau-mGFP mice were given 79mg/L clemastine for 7 days in their drinking water before perfusion-fixation after 21 days for immunohistochemical analysis. Differentiation in clemastine-treated animals did not differ from control animals in the cingulate cortex. p=0.07, unpaired two-tailed t-test; control: n=9, clemastine: n=4. Data are shown as mean ±SEM, with grey dots indicating individual animals.

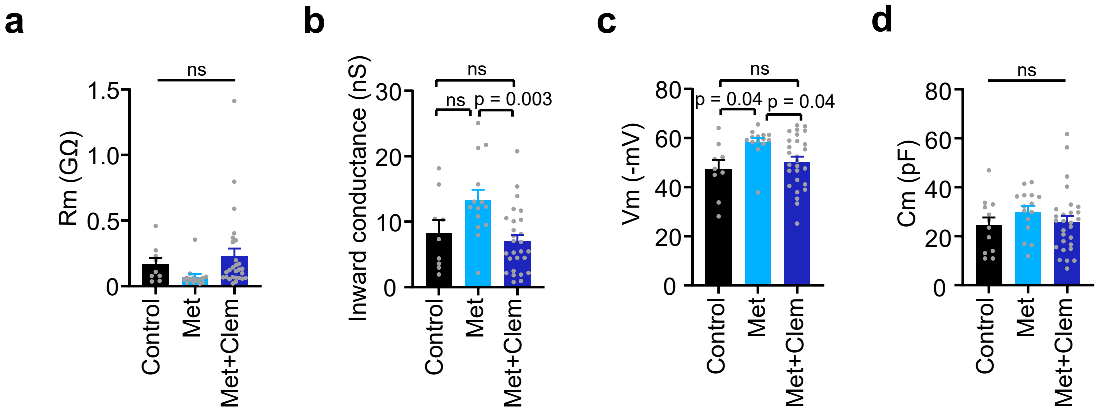

Supplementary Figure S4. Metformin alters passive membrane properties in OPCs
a Membrane resistance (Rm) does not differ with metformin or metformin+clemastine treatment in adult cortical OPCs. p=0.1, one-way ANOVA, control: n=9, metformin: n=14, metformin+clemastine: n=27. **b** Inward rectifying K+ conductance (inward conductance) was increased by metformin treatment alone, but not by metformin+clemastine treatment. p=0.004, one-way ANOVA, control: n=9, metformin: n=14, metformin+clemastine: n=27. p values on the bar graph are from Holm-Bonferroni post-hoc tests. **c** Metformin treatment alone hyperpolarized OPCs, but metformin+clemastine administration did not alter resting membrane potential (Vm). p=0.007, one-way ANOVA, control: n=9, metformin: n=14, metformin+clemastine: n=27. p values on the bar graph are from Holm-Bonferroni post-hoc tests. **d** Membrane capacitance (Cm), a proxy for cell surface area, did not differ between conditions. p=0.4, one-way ANOVA, control: n=12, metformin: n=15; metformin+clemastine: n=28.
